# Short Term Culture of Human Mesenchymal Stem Cells with Commercial Osteoconductive Carriers Provides Unique Insights into Biocompatibility

**DOI:** 10.3390/jcm2030049

**Published:** 2013-08-19

**Authors:** Matthew B. Murphy, Richard K. Suzuki, Theodore T. Sand, Christopher D. Chaput, Carl A. Gregory

**Affiliations:** 1 Department of Cellular Therapies, Celling Biosciences, Austin, Texas 78701, USA; E-Mails: mmurphy@cellingbiosciences.com (M.B.M.); rsuzuki@cellingbiosciences.com (R.K.S.); tsand@cellingbiosciences.com (T.T.S); 2 Department of Orthopaedics, Scott and White Hospital, Temple, TX 76504, USA; E-Mail: cchaput@sw.org; 3 Institute for Regenerative Medicine, Scott and White Hospital, Texas A & M Health Science Center, Temple, TX 76502, USA

**Keywords:** mesenchymal stem cells, bone marrow stromal cells, bone void fillers, osteoinductive substrates, spinal fusion

## Abstract

For spinal fusions and the treatment of non-union fractures, biological substrates, scaffolds, or carriers often are applied as a graft to support regeneration of bone. The selection of an appropriate material critically influences cellular function and, ultimately, patient outcomes. Human bone marrow mesenchymal stem cells (BMSCs) are regarded as a critical component of bone healing. However, the interactions of BMSCs and commercial bone matrices are poorly reported. BMSCs were cultured with several commercially available bone substrates (allograft, demineralized bone matrix (DBM), collagen, and various forms of calcium phosphates) for 48 h to understand their response to graft materials during surgical preparation and the first days following implantation (cell retention, gene expression, pH). At 30 and 60 min, bone chips and inorganic substrates supported significantly more cell retention than other materials, while collagen-containing materials became soluble and lost their structure. At 48 h, cells bound to β-tricalcium phosphate-hydroxyapatite (βTCP-HA) and porous hydroxyapatite (HA) granules exhibited osteogenic gene expression statistically similar to bone chips. Through 24 h, the DBM strip and βTCP-collagen became mildly acidic (pH 7.1–7.3), while the DBM poloxamer-putties demonstrated acidity (pH < 5) and the bioglass-containing carrier became basic (pH > 10). The dissolution of DBM and collagen led to a loss of cells, while excessive pH changes potentially diminish cell viability and metabolism. Extracts from DBM-poloxamers induced osteogenic gene expression at 48 h. This study highlights the role that biochemical and structural properties of biomaterials play in cellular function, potentially enhancing or diminishing the efficacy of the overall therapy.

## 1. Introduction

The practice of medicine in orthopedic surgery has included bone regeneration since the earliest bone grafts and bone marrow transfers nearly a century ago [1,2,3,4,5,6]. More recently, the disciplines of tissue engineering and regenerative medicine have permitted systematic evaluation of combinations of scaffolds or graft materials with stem and progenitor cells (either implanted with the graft or [7,8] recruited naturally from nearby tissues) [9,10,11,12], and growth factors for the purpose of directed tissue formation. Accordingly, the literature now contains a vast number of examples of successful cell-based therapies in bone tissue, albeit most in an experimental setting. The overall consensus of these studies is that regardless of the cell source employed, it is the cells that are directly responsible for tissue formation, not the scaffold, drug, or surgical instrumentation. In many instances, scientists and clinicians have attempted to supplement or replace cells by administration of potent growth factors, including bone morphogenetic proteins (e.g., BMP-2, BMP-7) [11,12]. Of the numerous cell sources that have been tested for bone tissue engineering, mesenchymal stem or stromal cells (MSCs) have received the most attention. MSCs are multi-potent progenitors of connective tissue such as bone, cartilage, muscle and adipose [13,14,15,16,17,18,19,20,21,22,23,24,25,26,27], and can be harvested from bone marrow, periosteum, synovium, muscle, fat, and other adult tissues [28,29,30,31,32,33]. Of the countless studies performed exploring the potential for MSCs for the repair bone tissue, some have demonstrated that they have remarkable efficacy in the absence of a scaffold [34,35,36,37,38,39] but the general consensus is that a scaffold may be required for confinement of MSCs at the site of injury during initial stages of healing [1,7,10,26,37,38,40,41,42]. 

Natural graft materials include autograft (tissue harvested from the patient) [6,43,44], allograft (tissue transplanted from another donor, usually a cadaver) [3,4,6], or processed tissue/proteins from cadaveric humans or animals [3]. Autograft is considered the “gold-standard” in fracture repair and spinal fusion, usually requiring the harvest of a portion of the iliac crest [43,44]. This strategy has greatly decreased in popularity and use, as it leads to donor-site morbidity (necrosis at the harvest site) and enduring pain to the patient [45,46]. Allograft products present some risk of disease transmission, inflammation and immune response to the foreign tissue [2,46]. Due to these shortcomings, synthetic biomaterials have been developed comprised of ceramics, proteins, peptides, carbohydrates, and polymers [1,3,7,10,47]. Synthetic materials can be designed to achieve the desired biological and mechanical properties, degradation or bioresorption rates, and tissue integration based on specific applications [3,10]. For orthopedic purposes, synthetic materials consist of calcium phosphates (including hydroxyapatite (HA) and β-tricalcium phosphate (βTCP)), collagen, protein, or hydrophilic polymer-based foams and gels, and hydrophobic polymers (e.g., poly(l-lactic-co-glycolic acid), polyurethanes, poly(propylene fumarate)). Calcium phosphate-based materials are advantageous as they offer natural osteoinductive cues through their ionic crystalline structure while providing a framework to build new tissue upon that is slowly resorbed and remodeled. Gan has reported successful spinal fusions in a human clinical study using autologous BMSCs augmented with βTCP granules [48].

The use of graft materials in spine and orthopedic markets has primarily focused on bone-derived or bone-like substrates including allograft bone chips and powderized bone, demineralized bone matrix (DBM, acidified allograft bone), calcium phosphate particles and cements, and collagen sponges [47,49]. Commercially marketed graft materials have come to be known as carriers for their ability to “carry” cells and proteins with them. More accurately, they are also referred to as scaffolds and substrates, as they provide a foundation for cells to lay down new extracellular matrix (ECM) and build mature tissue. Graft materials vary greatly in their form, origin, and biochemical properties. Substrates used in fusions, non-union skeletal defects, and as bone void fillers are often selected by surgeons based on appearance or handling properties rather than their effect on co-implanted or endogenous stem and progenitor cells. In fact, the nature of the interactions between primary human osteoprogenitor cells and most bone tissue substitutes are poorly understood. Another critical, but poorly understood characteristic of common bone substitutes is their degradation rate and consequent release of factors that affect the local microenvironment.

To better understand the effects of graft materials on osteoprogenitor cell function, primary human bone marrow MSCs (BMSCs) were seeded onto several commercially available substrates and incubated in a surgically-relevant duration for up to 48 h. Viable BMSC adhesion was measured over at 30 min, 1 h and 48 h by quantitative real-time reverse transcriptase polymerase chain reaction (qRT-PCR). The expression of bone-related genes was also quantified by RT-PCR at the conclusion of 48 h culture. During this period, the physical state of the material and pH also was also monitored. In another series of experiments, we evaluated the effect of agents released from three injectable poloxamer-based demineralized bone matrix preparations on proliferation and osteogenic gene expression. From these studies, we found that bone substitutes vary widely in their ability to bind BMSCs and activate common osteogenic markers. While some matrices facilitate BMSC binding and enhance osteogenic markers, others fared poorly in physiologically-buffered serum-containing media, losing their three dimensional form within a few minutes. In some cases, the materials also caused substantial changes in the pH of media that affected the viability of cells. Of the injectable materials tested, BMSCs cultured with soluble extracts of all 3 substances reduced cell expansion but two of them enhanced expression of osteogenic markers. From the *in vitro* data presented here, we predict that while some bone substitutes have some positive attributes with respect to cell binding and osteogenic stimulation, many have potentially undesirable effects that should be seriously considered before utilizing clinically.

## 2. Materials and Methods

### 2.1. Cell Culture

Human BMSCs preparations were generated from a healthy human donor as previously described [19]. Briefly 2 mL of iliac crest bone marrow was processed by discontinuous density-gradient centrifugation and 20 million cells of the mononuclear fraction were cultured in complete culture media (CCM) consisting of alpha-minimal essential media (Invitrogen, Carlsbad, CA, USA) containing standard concentrations of penicillin and streptomycin (Invitrogen) and 20% (v/v) fetal bovine serum (FBS, Atlanta Biologicals, Norcross, GA, USA). Cells were incubated at 37 °C with 5% (v/v) CO_2_ with media changes every three days until a density of about 2000 plastic adherent cells per cm^2^ was attained. Cells were re-plated at 100 per cm^2^ and allowed to grow for 7–9 doublings before re-passage. The identity of the cell preparations was confirmed by differentiation to mineralizing osteoblasts, adipocytes and chondrocytes and surface phenotype as described previously [19,36,37]. Cryopreserved vials of 1 million BMSCs were stored in liquid nitrogen at passage 2 for this study.

### 2.2. Orthopedic Substrates

Substrates investigated in this study include allograft cancellous bone chips (Allograft Cancellous Bone, IsoTis OrthoBiologics, Irvine, CA, USA), powdered cancellous bone (Cortical-Cancellous Bone Powder, Bone Bank Allografts, San Antonio, TX, USA), DBM strip/sponge (Accell TBM, Integra, Plainsboro, NJ, USA), DBM putty (OrthoBlast II, Citagenix, Laval, QC, Canada), DBM poloxamer-putty (Accell Connexus and OsteoSurge, Integra, Plainsboro, NJ, USA), collagen sponge (DuraGen, Integra, Plainsboro, NJ, USA), βTCP (SBM Bio-1, SBM, Winchester, MA, USA), βTCP-collagen morsels (Mozaik, Integra, Plainsboro, NJ, USA), HA-collagen (NanOss, Pioneer Surgical, Marquette, MI, USA), HA-collagen-bioactive glass (Vitoss BA, Orthovita, Malvern, PA, USA), 60:40 βTCP-HA granules (CymbiCyte, Celling Biosciences, Austin, TX, USA) and porous HA granules (Solum IV, under development by Celling Biosciences, Austin, TX, USA). The porous HA granules have a surface area of 7 m^2^ per gram of material according to the manufacturer’s specifications.

### 2.3. Cell Retention

BMSCs (500,000 cells in 1 mL CCM) were mixed with 0.5 mL of each substrate at a ratio of 1:2 (cell suspension to substrate, v/v). The mixture was incubated 30 and 60 min at 37 °C with inversion every 15 min or 48 h at 37 °C with 5% (v/v) CO_2_. After incubation, excess media was carefully removed and replaced by 2 mL of cation-free phosphate buffered saline (PBS). Samples were centrifuged at 400× *g* for 5 min, the supernatant was removed, and the process was repeated 2 more times to remove loosely-attached cells. To prevent substrate constituents from interfering with the RNA extraction and final purity, cells were dissociated from matrices using a trypsin/ethylene-diamine-tetraacetic acid dissociation cocktail (0.25% Trypsin-EDTA, Gibco, Grand Island, NY, USA) for 7 min at 37 °C. Cells were recovered by centrifugation followed by extraction of total RNA (High-Pure mRNA extraction kit, Roche Diagnostics, Indianapolis, IN, USA). One tenth of the RNA was then used to generate a mixture of oligo-dT and random-hexamer primed cDNA using a kit (Super-Script III Kit, Invitrogen). The original number of recovered and viable cells was determined by measuring the copies of glyceraldehyde phosphate dehydrogenase (GAPDH) cDNA using qRT-PCR. For this purpose, one fifth of the cDNA was amplified with a previously described primers and conditions [50] (Table 1) with fast SYBR-Green master mix (Applied Biosystems, Foster City, CA, USA) on a Bio-Rad C1000 thermocycler fitted with a CFX96 Real Time System (Bio-Rad, Hercules, CA, USA). Cycle thresholds (Ct) were compared with known standards run in parallel and converted to the original number of cells per sample. The experiment was performed in quadruplicate (*n* = 4) for each substrate and data were expressed as means with standard error of the mean (SEM).

**Table 1:** RT-PCR primer sequences for glyceraldehyde phosphate dehydrogenase (GAPDH), Runx2, alkaline phosphatase (ALP), and collagen I.

Gene	Forward primer sequence	Reverse primer sequence	Notes
GAPDH	CTCTCTGCTCCTCCTGTTCGAC	TGAGCGATGTGGCTCGGCT	55 °C anneal [49]
Runx2	GCAAGGTTCAACGATCTGAGA	TCCCCGAGGTCCATCTACTG	55 °C anneal [51]
ALP	GACCCTTGACCCCCACAAT	GCTCGTACTGCATGTCCCCT	55 °C anneal [52]
Collagen I	GAACGCGTGTCATCCCTTGT	GAACGAGGTAGTCTTTCAGCAACA	50 °C anneal [50]

### 2.4. Osteogenic Gene Expression for Mineralized Substrates

BMSCs and mineralized substrates (cancellous bone chips, βTCP-HA, and porous HA) were incubated as described above in complete medium with 20% FBS for 48 h in a 37 °C incubator with 5% CO_2_. Copy DNA was prepared as described, and the relative expression levels of collagen I [51], alkaline phosphatase (ALP) [51] and runt-related transcription factor 2 (Runx2) [53] was measured using previously described primers and conditions (Table 1). The genes were chosen to reflect early and intermediate markers of osteogenic differentiation that would be expected to be up-regulated during adherence to substrates over a 48 h period. One microgram of cDNA was added to each reaction, and expression levels relative to monolayer BMSCs were calculated using the 2-delta-delta Ct method, normalizing to GAPDH expression [54].

### 2.5. BMSC Expansion and Osteogenic Gene Expression with DBM and DBM-Poloxamer Putties

Soluble substrates (2 mL, DBM putty and DBM poloxamer-putty) were dissolved in 10 mL complete medium by incubation for 15 h at 4 °C with frequent inversion. Due to substantial acidity, the extracts were diluted a further 1:10 with medium until the pH reached 7.4. For proliferation studies, cultures of BMSCs (*n* = 3 per measurement) were established in 6-well 9.5 cm^2^ tissue culture plates (Invitrogen) by plating at 150 cells per cm^2^ in DBM conditioned media. Media was changed every 2 days and as a negative control, monolayer BMSCs were cultured in complete medium without DBM conditioning. At day 1, 2, 4 and 8 the cells were recovered by trypsinization and counted by Cy-Quant (Invitrogen, Grand Island, NY, USA) fluorescence incorporation assay as previously described [55]. For expression studies, cultures were established in 55 cm^2^ plates in the same manner but allowed to proceed for eight full days with media changes every two days. After 4 and 8 days, cells were recovered by trypsinization, total RNA was extracted and expression levels of Runx2, ALP, and type I collagen was measured. The experiment was performed in triplicate (*n* = 3) for each substrate. Data were calculated using the 2-delta deltaCt method, normalizing to GAPDH expression and expressed as fold-changes relative to control cultures not conditioned with DBM extract.

### 2.6. Resorption and pH

Each of the substrates was incubated at room temperature for 24 h to observe physical changes and dissolution and measure the pH after hydration with a buffered solution. In separate 15 mL vials, each material (2 cc) was submerged in 3 mL human plasma or PBS. Plasma was prepared by centrifuging whole blood (South Texas Bood and Tissue Center, San Antonio, TX, USA) for 10 min at 1000× *g*. Plasma and PBS-only tubes were prepared and measured at each time point as a control. The tubes were shaken gently every 15 min. The pH of each solution was measured using an Orion-4 pH probe (Thermo Scientific, Waltham, MA, USA) incrementally for 24 h. The experiment was run in triplicate for each substrate.

### 2.7. Statistics

Data were analyzed by one sided analysis of variance (ANOVA) with Tukey or Dunnett’s post-testing using commercially available software (GraphPad Prism version 5.00 for Windows, GraphPad Software, San Diego, CA, USA).

## 3. Results

### 3.1. Cell Retention

**Figure 1:**
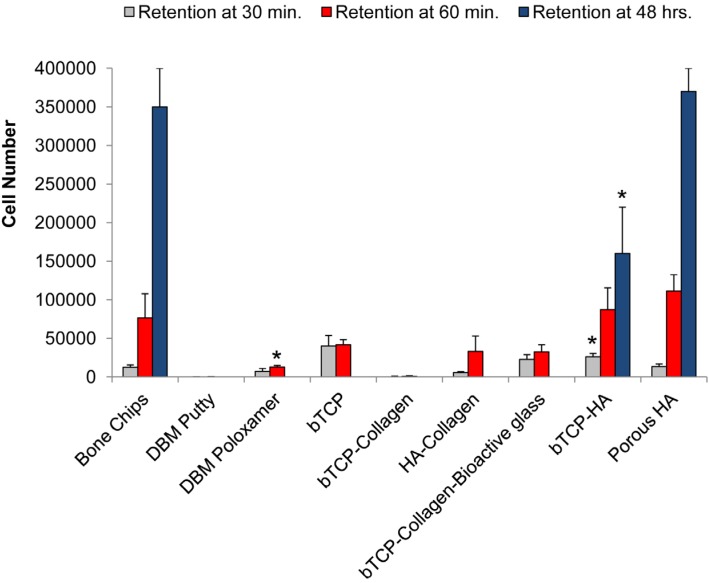
Bone marrow stromal cell retention on insolubilized substrates after 30 min, 60 min, and 48 h of incubation at 37 °C. Error bars represent standard error of the mean (*n* = 4 per substrate). Asterisk denotes significant difference (* *p* < 0.05) against the appropriate bone chip measurements by ANOVA and Dunnett post test.

**Figure 2:**
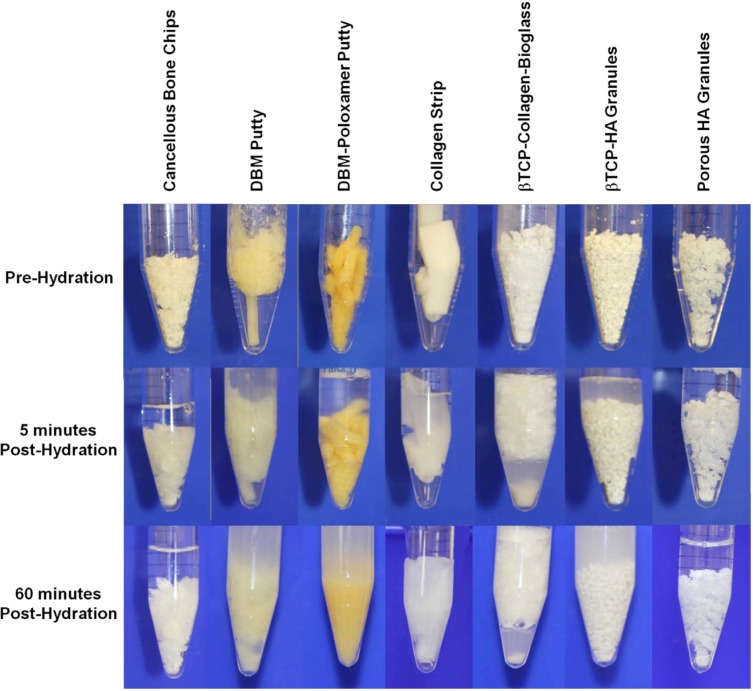
Cancellous bone chips, demineralized bone matrix (DBM) putty, DBM-poloxamer putty, collagen, βTCP-collagen-bioactive glass, βTCP-HA granules, and porous HA granules (left to right) prior to hydration with phosphate buffered saline (top row), 5 min after hydration (middle row), and 60 min after hydration (bottom row).

The kinetics of BMSC binding and retention is shown in Figure 1. After 30 min of culture, DBM and collagen-based materials had significantly dissolved into the media leaving about 30% of the original solid volume to collect for measurement and consequently, cell recovery was low. The structural degradation of substrates before and after hydration is demonstrated in Figure 2. Synthetic materials made of βTCP, βTCP-HA, and porous HA retained their structural integrity over the entire culture period, and rapidly bound more BMSCs over a 30 min period when compared to cancellous bone chips. After one hour, cell counts on βTCP-HA and porous HA granules were comparable to bone chips and significantly greater than the other substrates examined by the prescribed methods. After 48 h of culture, the bone chips, porous HA and to a lesser extent, βTCP-HA all had significant numbers of cells associated with them, suggesting that these materials could sustain stable constructs. In contrast, stable cultures of BMSCs on βTCP could not be established in our culture system, and the cell retention was diminished to below detectable levels after 24 h.

### 3.2. Osteogenic Differentiation

**Figure 3:**
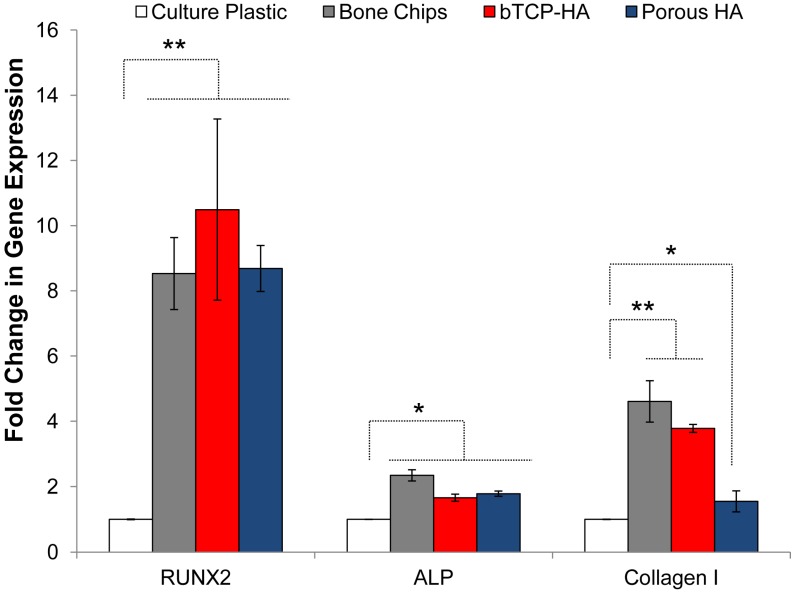
Relative expression of Runx2, alkaline phosphatase (ALP), and type I collagen as a fold increase over tissue culture plastic by cells incubated with cancellous bone chips, βTCP-HA, and porous HA granules after 48 h of incubation at 37 °C. Error bars represent standard error of the mean (*n* = 4 per substrate). Asterisks denote significant difference (* *p* < 0.05, ** *p* < 0.01) by ANOVA and Tukey post test.

To evaluate the short-term differentiation potential of BMSCs bound to substrate materials, the transcription of the early osteogenic transcription factor Runx2, intermediate markers ALP and collagen I were measured by quantitative RT-PCR (Figure 3). Experiments were performed on those materials that could sustain stable cultures (bone chips, βTCP-HA and porous HA) and results were compared to expression levels on tissue culture plastic. All of the osteogenic materials induced modest upregulation of transcription of ALP (1.5–2 fold) and collagen I (1.5–5 fold) when compared to tissue culture plastic but expression of Runx2 was substantially up-regulated by culture (8–10 fold). 

### 3.3. BMSC Expansion and Osteogenic Gene Expression with DBM and DBM-Poloxamer Putties

**Figure 4:**
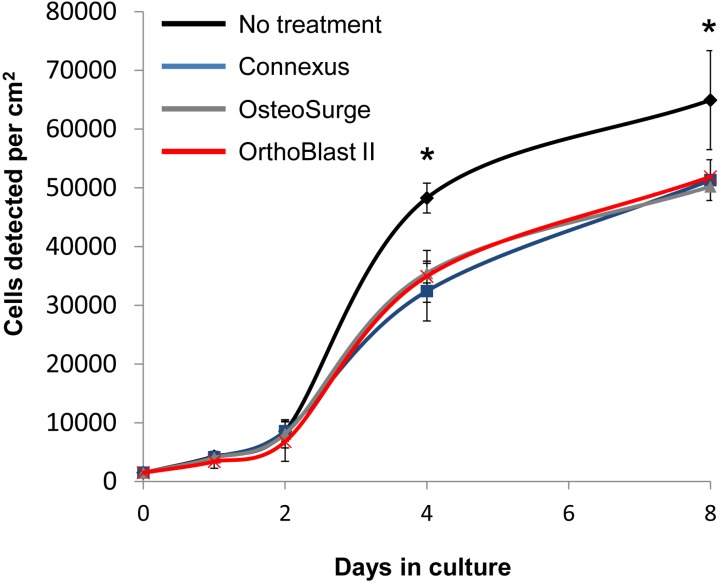
Cell growth curves for BMSC cultured in standard medium or 1:10 diluted extracts of DBM or DBM-poloxamer putties through 8 days (*n* = 4 per substrate). Asterisk denotes significant difference (* *p* < 0.05) against the no treatment control by ANOVA and Dunnett post test.

**Figure 5:**
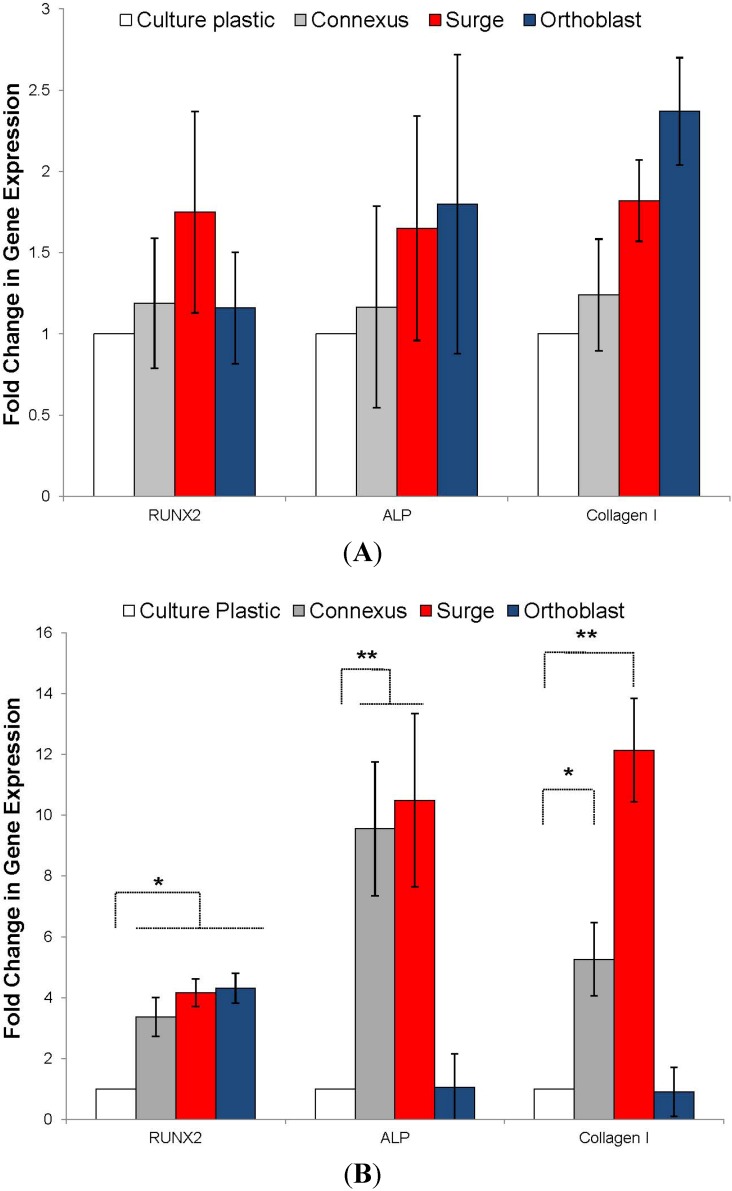
Relative expression of Runx2, alkaline phosphatase (ALP), and type I collagen as a fold increase over tissue culture plastic by cells cultured in 2D with 1:10 diluted extracts from DBM and DBM-poloxamer putties after four days (**A**) and eight days (**B**) of incubation at 37 °C. Error bars represent standard error of the mean (*n* = 4 per substrate). Asterisks denote significant difference (* *p* < 0.05, ** *p* < 0.01) by ANOVA and Tukey post test.

Three injectable putty preparations examined in this study for support of BMSC proliferation and differentiation; two DBM poloxamer-putty preparations (Accell Connexus and OsteoSurge, Integra, Plainsboro, NJ, USA) and one DBM putty (OrthoBlast II, Citagenix, Laval, QC, Canada) were studied. Because the materials partially dissolved in media, it was not possible to perform a conventional cell binding assay. We therefore prepared filtered extracts of the samples in growth media by incubation at 4 °C for 15 h. The DBM extract media was incubated with 2D monolayer culture BMSCs. Initial observations indicated that the 2 poloxamer-based putties caused substantial acidity of the extracts (all cells died within 12 h), but this could be buffered by 1:10 dilution in fresh medium. Upon monolayer culture in the presence of the extracts it was apparent that all three samples caused a minor reduction in cell recovery after 4 and 8 days (Figure 4), but the absence of morphological signs of cell death suggested that the effects were due to cell-cycle inhibition rather than necrosis or apoptosis. After 4 and 8 days of culture, the relative transcription levels of Runx2, ALP and collagen I were measured by real time RT-PCR. At day 4, transcriptional levels of all three genes did not significantly differ from the control media. However, after eight days of culture, extracts from all putties caused a modest increase in Runx2 transcription (3–4 fold) when compared with untreated controls (Figure 5). Interestingly, only the DBM-poloxamer extracts caused an appreciable increase in ALP and collagen I at 8 days, suggesting that osteogenesis had progressed further in these cultures. Taken together, these data suggest that although modestly detrimental to BMSC expansion, extracts from the DBM and DBM-poloxamer materials had a positive effect on osteogenesis *in vitro* if the acidic properties can be maintained within the physiological pH range.

### 3.4. Resorption and pH

The plasma and PBS solution pH of each substrate through 48 h is demonstrated in Table 2. Most substrates remained within the normal physiologically neutral range (pH 7.3–7.5) for the duration of the investigation and with little variation after the initial 5 min in solution. The most acidic products were the DBM strip (pH < 7.2) and the DBM poloxamer-putty (pH < 4.9). The Βtcp-collagen-bioactive glass material elicited a basic response (pH > 10). 

**Table 2:** Average pH values of substrates buffered in human plasma over 48 h (pH values in phosphate buffered saline in parentheses).

Substrate	1 min	5 min	30 min	60 min	24 h	
βTCP-Collagen-Bioactive glass	8.47 (6.73)	9.67 (7.33)	9.68 (7.95)	9.57 (9.16)	10.09 (10.49)	Highly Basic
DBM putty	7.66 (7.11)	7.75 (7.34)	7.72 (7.55)	7.62 (7.52)	7.92 (7.53)	Within Physiological Range
Cancellous bone chips	7.61 (7.21)	7.65 (7.17)	7.59 (7.17)	7.47 (7.17)	7.75 (7.14)	
Collagen	7.66 (7.33)	7.62 (7.40)	7.49 (7.30)	7.47 (7.39)	7.15 (7.35)	
Porous HA	7.54 (7.23)	7.56 (7.24)	7.50 (7.33)	7.42 (7.39)	7.74 (7.36)	
βTCP-HA	7.54 (7.20)	7.73 (7.25)	7.48 (7.20)	7.39 (7.21)	7.80 (7.22)	
HA-Collagen	7.44 (6.94)	7.47 (6.98)	7.37 (7.06)	7.32 (7.08)	7.61 (6.85)	
βTCP-Collagen	7.32 (7.01)	7.38 (6.91)	7.44 (6.84)	7.29 (6.71)	7.65 (6.62)	Slightly Acidic
DBM strip	7.42 (6.70)	7.34 (6.38)	7.24 (6.39)	7.14 (6.46)	5.44 (6.27)	
DBM poloxamer	6.23 (6.74)	6.13 (6.07)	5.61 (5.06)	4.87 (4.66)	4.95 (4.42)	Highly Acidic

## 4. Discussion

In an *in vitro* setting, cell retention was measured on 9 different commercially available carrier substrates at 30 and 60 min, and 48 h (Figure 1). A cell suspension was added to the substrate at a 1:2 volumetric ratio. The first observation was the dissolution of DBM and collagen-containing materials. DBM carriers are synthesized by the acidification of allograft bone with hydrochloric and/or citric acid to remove mineral, preserving a matrix of organic proteins, consisting primarily of primarily collagen fibrils [56,57,58]. Most collagen and collagen-composites are synthesized by the lyophilization (freeze-drying) or precipitation of a solubilized collagen derived from acidified bovine or porcine tissues [59,60]. For DBM and collagen substrates, the introduction of water, saline, blood, or other fluids hydrates the material, making it softer by dissolving a portion of the protein matrix. The hydration of dry collagen matrix can lead to physical constriction of the material, decreasing the average pore size and thereby limiting its accessibility to cells and blood vessels. As the proteins dissolve, the loss of available binding surface area may render the material ineffective as a scaffold to bridge a bony defect. The dissolution of DBM and collagen-containing carriers made evaluation of cell retention beyond early time points impossible. Two of the collagen-containing materials demonstrated cell retention at 30 min comparable to bone chips and the calcium phosphate materials. However, as the materials resorbed into the media, there was little or no physical material to collect for cell quantification. This study aimed to identify the number of cells resident on an implant material through 48 h to provide researchers and physicians insight into the fate of BMSCs. If the cell-bound portions of the materials are resorbed between 60 min and 48 h *in vitro*, there is likely some corollary to *in vivo* material resorption and potential cell loss (migration away from the implant site or death). The provision of a stable ECM is essential for sufficient retention of cells at the implant site [37]. The βTCP/HA and HA substrates maintained their mass and geometry in solution through the duration of the study and achieved increasing cell counts with time. The successful binding between cells and ceramic materials can be attributed initially to electrostatic interactions of membrane proteins with calcium and phosphate ions on the granules’ surface. The increased cell counts from 30 to 60 min are related to gradual binding of cells, however, the drastic gain in cell number at 48 h is undoubtedly due to cell proliferation on the substrates as an extracellular matrix is established on the surface of the material. Porous HA granules possess more than 7 m^2^ of surface area per gram, at least six-fold increase over typical calcium phosphate particulates. The increased surface area permitted greater cell proliferation and spreading, which demonstrated the highest cell counts at the later time point. 

The second observation upon addition of cell suspension to the substrates was an immediate change in media color for several samples. The culture media used in these experiments contained phenol red pH indicator dye, which is responsive to pH changes associated with depleted nutrients and accumulated waste products. The DBM strip and DBM poloxamer-putty caused the media to change from red to yellow over the first 30 min of incubation, indicating a drop in pH and the acidity of the solution. In contrast, the bioactive glass material rapidly turned the media from red to purple (less than one minute), meaning the solution had become highly basic. This phenomenon was most likely to account for the exceptionally low cell counts for these materials, given that the pH was outside the physiological range for BMSC viability. To test this hypothesis, the experiment was repeated with human plasma and a buffered saline solution (PBS) rather than cell medium, and the pH was closely monitored with an electronic pH probe (Table 2). DBM poloxamer-putty decreased the solution pH below 5, while the DBM strip and βTCP-collagen substrates produced a less acidic effect. Although mild levels of transient acidity is tolerable to cells and is common with inflammation and infection, decreased pH can alter the chemical characteristics of many proteins and drugs, reducing their efficacy. Prolonged exposure to acidic conditions can result in apoptosis and tissue necrosis as well as inhibition of hydroxyapatite deposition during bone remodeling [61]. Conversely, the bioactive glass carrier achieved a solution alkalinity (pH 10) that is known to adversely affect cell viability *in vitro*, and thus, was likely the cause of very poor cell recovery. These results were surprising given that numerous favorable reports of bioglass and its application in bone engineering [62,63]. In partial explanation of this disparity, simulation studies have suggested that the hydroxyl moieties released through hydrolysis of the glass constituents do exist, but they are rapidly re-sequestered to the surface of the glass [64]. Although this could account for the reduced effects of alkalinity in some situations, the solution alkalinity detected in this study was approximately 300 times higher than physiological levels. Although surgery sites may be slightly acidic, the basicity of this bioglass is orders of magnitude higher. The bioglass components of this substrate were more easily and quickly dissolved than sintered or conjugated analogous materials. While the buffering capacity of blood and the body may negate long term deleterious effects of materials with non-ideal pH, short term incubation of these materials outside the body during graft preparation may impact cells’ viability and their therapeutic effects.

It is known that the biological, chemical, and physical properties of the graft materials directly influence the physiology of osteoblast progenitors. For example, nano-scale topological features such as porosity have influenced differentiation mechanisms of attached MSCs [65,66,67] and molecules on the surface may directly modulate receptors or achieve this indirectly through bound plasma proteins, growth factors, or other molecules. In the present study, we observed that BMSC adherence to bone chips, βTCP-HA, and porous HA increased Runx2 expression by 8.5, 10.5, and 8.7-fold when compared to tissue culture plastic monolayer controls. Runx2 is an early-stage transcription factor that activates osteoblastic differentiation [68] and is highly indicative of commitment to an osteoblastic lineage. This level of Runx2 up-regulation was unexpectedly high for a 48 h time point without dexamethasone or bone morphogenic administration supporting the critical role of the solid substratum in addition to biochemical stimulation. On the other hand, the expression of ALP was only moderately increased for these substrates (2.3, 1.7, and 1.8-fold, respectively) but this protein is usually upregulated by BMSCs after more than 48 h or osteogenic stimulation under standard conditions [55]. Bone chips triggered a greater up-regulation of type I collagen than either synthetic substrate (4.6-fold over culture plastic compared to 3.8-fold for βTCP-HA and 1.5-fold for porous HA). We hypothesize that this effect is partially due to existing collagen and ECM proteins within the bone chips and absent from the ceramic particles that may promote further ECM remodeling and synthesis. 

It was hypothesized that the DBM putties would enhance BMSC proliferation and differentiation given the reported effects of bone extracts on osteoprogenitor function [58,69,70,71,72,73]. However, their rapid resorption and the acidity associated with their fabrication proved incapable of maintaining cell growth *in vitro*. After dilution of DBM and DBM-poloxamer extracts at 1:10 in standard media, cells were able to proliferate on culture plastic at retarded rates. These reduced growth rates may be attributed to cell cycle changes associated with differentiation as well as the toxicity of the extracted acidic byproducts. After 48 h in culture, the diluted DBM and DBM-poloxamer extracts induced expression of osteogenic genes, validating their osteogenic properties once the acidic effects are mitigated. 

## 5. Conclusions

Several commercially available orthopedic substrates were examined for their interactions with BMSCs within 48 h to mimic cell response after surgical preparation of graft materials and implantation in orthopedic or spinal fusion procedures. DBM and collagen-based substrates quickly resorbed upon hydration, rendering them incapable of cell retention or serving as a lasting scaffold for tissue regeneration *in vivo*. The DBM and DBM-poloxamer materials also exhibited a release of acidic factors after hydration, preventing cell proliferation without dilution of the DBM extracts. After adjustment of media pH, however, the DBM-poloxamer substrates did elicit an osteogenic effect on plastic-bound cells in terms of Runx2, ALP, and collagen I expression. The DBM putty only increased Runx2 expression compared to standard controls. Cancellous bone chips, βTCP-HA, and porous HA granules supported cell retention and 3D proliferation through the 48 h of observation and induced significantly greater expression of Runx2, ALP, and collagen I compared to controls.

The application of mesenchymal stem and progenitor cells is a vital component for regenerative medicine therapies. However, the choice of substrate accompanying the cells can facilitate their success, inhibit their functionality, or cause their premature death depending on the material’s structure, composition, and byproducts. The primary purpose of biomaterial scaffolds is to provide a substrate for cell adhesion and proliferation that guides the growth of new tissue. In non-union fracture and spinal fusion procedures, substrates that quickly dissolve cannot perform either function. A lack of consideration or understanding of cell-substrate interactions may negate the regenerative potential of cell therapies and lead to undesired clinical outcomes. 
